# Obsessive compulsive disorder comorbidity in DBA

**DOI:** 10.1186/1745-0179-4-6

**Published:** 2008-03-10

**Authors:** Stefano Pallanti, Sara Masetti, Silvia Bernardi, Alice Innocenti, Mariana Markella, Eric Hollander

**Affiliations:** 1 Department of Psychiatry, The Mount Sinai School of Medicine, New York, NY, USA; 2 Department of Psychiatry, Università degli Studi di Firenze, Florence, Italy; 3 Istituto di Neuroscienze, Florence, Italy

## Abstract

Diamond-Blackfan Anemia (DBA) is a congenital erythroid aplasia characterized as a normochromic macrocytic anemia with a selective deficiency in red blood cell precursors in otherwise normocelullar bone marrow. DBA is known to be associated with mental retardation and learning disabilities. Although comorbidities with other psychiatric conditions have not been reported in the existing literature, we report in this paper a case of a DBA patient with previously undiagnosed comorbidity of obsessive compulsive disorder (OCD), successfully treated with sertaline 200 mg/day and valproic acid 600 mg/day. This case of comorbid presentation has clinical, therapeutic and pathophysiological implications. Given the difficulty of distinguishing among mental retardation, learning disabilities and OCD and the importance of precocious diagnosis in treating OCD especially since there are treatment methods interfering with anemia symptoms, physicians should adapt an adequate screening tool treating a child with DBA and comorbid mental disorder.

## Introduction

Diamond-Blackfan Anemia (DBA) is a congenital disease, charactereized by a defective erythroid progenitor maturation and is associated with physical malformations.

Majority of cases are sporadic and dominant with 10% of the patients demonstrating recessive inheritance.

Mutations in the gene encoding for ribosomal protein RPS19 (DBA1) have been found in 25% of patients with either the dominant or the sporadic traits [1,2].

It is noteworthy that these mutations are associated with mental retardation as well as with learning disabilities in DBA patients [1,3].

Somatic abnormalities have been found in 47% of the patients registered with the DBA Registry of North America [4,5].

Associated physical anomalies and growth retardation are common and outstanding even in patients with multifactorial etiology such as long term steroid treatment [3].

The combination of clinical and molecular findings suggests a contiguous gene syndrome with a gene focus for mental retardation and skeletal malformations.

Repetitive and stereotyped behaviors are as common as mental retardation and in some cases their manifestations reach the threshold for diagnosis of obsessive compulsive disorder (OCD) (according to the Diagnostic Statistical Manual of Mental Disease IV edition Text Revised DSM-IV TR criteria) [6].

In the following case we present a DBA patient with comorbidity of OCD. This case has tremendous significance due to the demonstration of the clinical and the pathophysiologic as well as therapeutic implications, involved in the assessment of behavioral abnormalities in DBA.

## Case presentation

L. is a 22 year old Italian male, diagnosed with DBA at the age of two. Since being diagnosed with DBA, L has been treated with monthly blood transfusions and subcutaneous injections of deferoxamine mesylate. Years later, he developed iatrogenic hepatitis due to multiple blood transfusions. Despite attending a special education program for children with learning disabilities, the patient has experienced major difficulties in carrying out daily activities since the age of six. He showed attention deficit at school, social isolation and, since the age of 12, verbal and motor repetitive behaviors, apparently cyclically worsening during mood instability episodes.

L was reluctant to speak about his repetitive behaviors. L's parents attributed their child's behaviours to the developmental disabilities. A standard psychiatric diagnosis was not reached, no treatment was established during childhood.

L' s grandfather was diagnosed with OCD (checking compulsions) in comorbidity with an Impulsive Control Disorder (Intermittent Explosive Disorders), his grandmother was depressed and alcohol addict.

At the age of sixteen the patient had an episode of herpetic encephalitis with symptoms of delirium and therefore, he was treated for two years with carbamazepine. One year later he was diagnosed with polyendocrinopathy of hypothyroidism, hypoparathyroidism and hypogonadism. The encephalitis process had no consequences. Neither mental state nor cognition alteration were reported following the episode.

At the age of twenty one repetitive behaviors increased in frequency to a level that required psychiatric attention and pharmacological management. Trying to address both mood symptoms and repetitive behaviors, a treatment with low doses of olanzapine and venlafaxine was established, with no improvement in symptoms and a strong deterioration of patient's anemia. L was hospitalized and a more thorough psychiatric assessment was conducted.

The patient is in the lower normal range of height (164 cm) and IQ (87). He complained of impulsive sexual and aggressive thoughts that were intrusive, repetitive and distressing. He also complained of compulsive behaviors and rituals, such as hoarding, arranging, ordering, preoccupations with symmetry, exactness, rewriting and doubting. Interrupting the patient while carrying out his rituals lead to violence. The patient had moderate insight of his illness.

He fulfilled the DSM-IV TR criteria for OCD and diagnosis was established by the Structural Clinical Interview for DSM-IV Axis I Disorders (SCID-I) [7]. In order to determine the severity level of obsessive compulsive symptoms, the Yale Brown Obsessive Compulsive scale (YBOCS) [8], a clinician rated 10 item scale, each rated from 0 (no symptoms) to 4 (extremely severe symptoms), was performed on him and revealed a score of 28, which corresponds to a severe form. A treatment with sertaline 200 mg/day (addressing OCD symptoms) and valproic acid 600 mg/day (with the aim of reducing the impulsive features linked to obsessions and according to its efficacy reported in treatment of DBA) [9] has been started.

In the meantime an MRI exam was done as well and it showed low signal areas due to accumulation of paramagnetic substances in the right temporal lobe and in the ventricular choroid plexus, asymmetric sphenoid sinus and hypoplastic pituitary gland. Sellar region and parasellar structures appeared in a regular pattern. No anomalies in encephalic parenchyma were demonstrated after contrast medium injection.

The patient was assessed 12 weeks after the administration of the YBOCS and demonstrated an improved total score of 15, which corresponds to a mild form of OCD with a reduction of more than 45% of the symptoms.

## Discussion

For the first time we have described DBA with comorbid OCD. The above described case could demonstrate heuristically valuable clinical, therapeutic and pathophysiological implications if more DBA patients with comorbid OCD would be screened by hematologists and therefore deserves further discussion.

There is a great importance in the assessment of obsessive-compulsive symptoms in DBA patients with mental or behavioral disturbances. Since OCD often goes undiagnosed in the presence of more pervasive disturbances [10], our report assumes a "Caveat" value.

Distinguishing between mental retardation, learning disabilities, Asperger Syndrome and OCD can be challenging, especially when treating children. Precocious diagnosis of OCD can make a tremendous difference in terms of evolutionary trajectory and improved life quality of patients and their families.

Pediatricians should bear in mind the possibility of OCD when treating DBA children with behavioralor learning disabilities even in the absence of other malformations.

It has been shown that when adequate screening tools were adopted in clinical disciplines other than psychiatry (for instance in dermatology and immunology), a larger than expected number of undiagnosed OCD patients was revealed [11].

Also, OCD is potentially linked to brain iron accumulation in DBA. Studies done in animals demonstrated that brain iron accumulation leads to damage of neuronal dopaminergic function.

Intranigral iron injection in rats have shown a detrimental effect on dopamine (DA) release and concentration in the caudate putamen (CPu) as well as selective decrease of striatal dopamine (95%), 3,4-dihydroxyphenylacetic acid serotogenic activity (82%), and homovanillic acid (45%) with related behavioral changes, characterized by increased repetitive and compulsive behaviors.

Thus, hemosiderosis might be contributing to psychiatric symptoms in DBA patients [12]. In fact, OCD symptoms may be linked to hemosiderin deposition in the brain and the pituitary gland, just as hypopituitarism has been shown to be linked to hemosiderin deposition in the pituitary as it was hypothesized previously by Berdel [13,14].

Moreover, since OCD has been related also to multiple regions of cortical thinning [15], the MRI imaging in our case of paramagnetic substance accumulation in the right temporal lobe, ventricular plexus and the hypoplastic pituitary gland is suggestive of the importance of neuroimaging assessment and recognition of complications caused by iron deposition due to long term blood transfusions in the management of DBA.

However a conclusion can not be drawn without a verification through larger studies on populations that have undergone blood transfusions at a young age.

Presenting this case report we have shown that OCD symptoms are treatable in DBA as effectively as in other conditions such as mental retardation [16] and Down Syndrome [17].

Despite the fact that some psychiatric medications have shown a worsening of the symptoms of anemia, SSRIs and valproate have been extremely beneficial and safe.

## Authors' contributions

SP established the treatment of the patient, conceived the case-report and drafted the manuscript, SM drafted the manuscript, SB drafted the manuscript, MM drafted the manuscript, AI collected information about the case and drafted the manuscript, EH drafted the manuscript. All authors read and approved the final manuscript.
